# Detection of Malicious Threats Exploiting Clock-Gating Hardware Using Machine Learning

**DOI:** 10.3390/s24030983

**Published:** 2024-02-02

**Authors:** Nuri Alperen Kose, Razaq Jinad, Amar Rasheed, Narasimha Shashidhar, Mohamed Baza, Hani Alshahrani

**Affiliations:** 1 Department of Computer Science, Sam Houston State University, Huntsville, TX 77340, USA; nxk022@shsu.edu (N.A.K.); raj032@shsu.edu (R.J.); axr249@shsu.edu (A.R.); nks001@shsu.edu (N.S.); 2 Department of Computer Science, College of Charleston, Charleston, SC 29424, USA; 3 Department of Computer Science, College of Computer Science and Information Systems, Najran University, Najran 61441, Saudi Arabia; hmalshahrani@nu.edu.sa

**Keywords:** malware, embedded systems, machine learning, intrusion detection, ARM cortex

## Abstract

Embedded system technologies are increasingly being incorporated into manufacturing, smart grid, industrial control systems, and transportation systems. However, the vast majority of today’s embedded platforms lack the support of built-in security features which makes such systems highly vulnerable to a wide range of cyber-attacks. Specifically, they are vulnerable to malware injection code that targets the power distribution system of an ARM Cortex-M-based microcontroller chipset (ARM, Cambridge, UK). Through hardware exploitation of the clock-gating distribution system, an attacker is capable of disabling/activating various subsystems on the chip, compromising the reliability of the system during normal operation. This paper proposes the development of an Intrusion Detection System (IDS) capable of detecting clock-gating malware deployed on ARM Cortex-M-based embedded systems. To enhance the robustness and effectiveness of our approach, we fully implemented, tested, and compared six IDSs, each employing different methodologies. These include IDSs based on K-Nearest Classifier, Random Forest, Logistic Regression, Decision Tree, Naive Bayes, and Stochastic Gradient Descent. Each of these IDSs was designed to identify and categorize various variants of clock-gating malware deployed on the system. We have analyzed the performance of these IDSs in terms of detection accuracy against various types of clock-gating malware injection code. Power consumption data collected from the chipset during normal operation and malware code injection attacks were used for models’ training and validation. Our simulation results showed that the proposed IDSs, particularly those based on K-Nearest Classifier and Logistic Regression, were capable of achieving high detection rates, with some reaching a detection rate of 0.99. These results underscore the effectiveness of our IDSs in protecting ARM Cortex-M-based embedded systems against clock-gating malware.

## 1. Introduction

Power optimization mechanisms have been widely adapted by today’s microcontroller designers to minimize the chip’s dynamic power consumption. Smart sensing technologies with limited energy resources (e.g., IoT platforms, health monitoring devices, energy monitoring systems, and radio communication modules) are widely integrated with a power-efficient microcontroller chipset based on an ARM Cortex M core to support low energy processing capabilities.

Microcontrollers based on the ARM Cortex M chipset become the ultimate choice for supporting low-cost and power-efficient processing on embedded systems. Low energy processing on the ARM Cortex M chipset is supported via the deployment of clock-gating methodology [1,2,3,4,5]. Clock-gating methodology is a hardware feature that enables dynamic activation/ deactivation of various subsystems on the chip to minimize dynamic power consumption. Examples of subsystems that are clock-gated include interrupt vector modules, USB, RTC, $I^{2}C$, UART, and RNG. Via chip configuration code running on the chipset during boot time, subsystems that are not used by the embedded system application can be deactivated to lower dynamic energy consumption.

Although hardware-based optimization techniques based on the clock-gating approach enable power-efficient processing, such techniques can be exploited by an attacker to target the subsystems’ availability during software execution. A recent study presented by A. Rasheed et al. (2021) [1] showed that embedded systems implemented with an ARM Cortex M microcontroller chipset are vulnerable to clock-gating-assisted malware attacks. The author introduced new malware threats that compromise the chipset configuration file during boot time by injecting malicious code. It aims at modifying the chip’s configuration parameters during the initialization process by disabling/enabling various subsystems on the chipset. For example, an attacker will be able to disable the RNG module on the chipset, impacting the reliability of cryptographic computations. Another example includes disabling serial communication ports on the chipset where sensor systems became unable to transmit and receive data. Several variants of the malware were presented in [1] and include Power Hungry, PIT-off, UART-killer, and $I^{2}C$ killer.

Signature-based approaches to malware detection in embedded systems are limited by their reliance on known malware signatures. Zero-day attacks and advanced malware variants that can modify their signatures make this approach inherently ineffective. The malware signature database must be constantly updated in signature-based systems in order to keep up with the evolving landscape of malware threats [6,7]. They are also less effective at detecting sophisticated attacks that exploit hardware vulnerabilities, such as clock-gating, which are difficult to detect with traditional signatures [8].

In contrast, the proposed Intrusion Detection Systems (IDSs) in this research leverage machine learning techniques to overcome these limitations. Our IDSs analyze power consumption data and employ advanced classification algorithms to detect anomalies and patterns indicative of clock-gating-assisted malware, regardless of the attack signature. With this approach, embedded systems can be protected against evolving threats by detecting zero-day attacks and sophisticated malware variants that exploit hardware features.

This paper proposes the development and implementation of two IDSs capable of detecting several variants of the clock-gating-assisted malware presented in [1]. Six IDSs leveraging machine learning approaches based on K-Nearest Classifier, Random Forest, Logistic Regression, Decision Tree, Naive Bayes, and Stochastic Gradient Descent have been trained and validated using a dynamic power dataset collected from the chipset during malware execution and under normal operation. To the best of our knowledge, this research effort introduces the first solution for detecting hardware-based malware via classifying and identifying malware code injection attacks that exploit vulnerabilities in the clock-gating mechanism of the ARM Cortex M chipset. Our proposed IDSs consider unique characteristics of the ARM-based embedded system power consumption data for detecting and classifying clock-gating-assisted malware effectively. During this effort, we proposed the development of reliable and efficient IDSs that can operate within the resource-constrained nature of embedded systems. Our proposed IDSs aim to overcome the limitations of signature-based approaches by utilizing machine learning techniques for hardware-based malware detection on embedded systems with ARM Cortex M chipset. The proposed IDSs implement machine learning algorithms that have the capacity to accurately detect and categorize zero-day malware on hardware. The proposed IDS is capable of combining anomaly-based recognition, misuse-based recognition, and specification-based recognition procedures [2].

Malware data samples including nonanomalous data samples obtained from previous research efforts conducted by Rasheed et al. (2021) [1] were utilized for model training, testing, and validation. Our main objective in this paper is the development of IDSs capable of classifying malware presence via analyzing power consumption data of the ARM Cortex M chipset during program execution. To test our proposed IDSs, multiple embedded systems were employed and deployed with various variants of clock-gating-assisted malware codes. To truly simulate the behavior of a system under malware threat, each system was incorporated with multi-sensor modules capable of capturing sensor data from the infected embedded platforms, including measurements of light, temperature, humidity, accelerometer, and pressure readings. Among the sensing unit and the primary chip, data from these sensors were sent utilizing the uart and $I^{2}C$ serial communication channels. In order to make use of the SIM module, the malware altered the bits of values in the system clock-gate management registers. For all variations of the suggested malware, malicious code injections were made into the “systemInit()” method. Intruders could access these registers during the boot-up process by inserting malicious code within the “systemInit()” function. During this research, our IDSs were tested against four distinct malware strains: Power Hungry, PIT-off, uart killer, and $I^{2}C$ killer.

The proposed IDSs employ various models on a power consumption dataset, including four types of clock-gating-assisted malware and normal clock-gating operations. Our main objectives in this research are the following:

- To highlight software threats and attacks against clock-gating techniques in embedded systems.
- To propose IDSs using machine learning models to identify and classify clock-gating-assisted malware correctly.
- To examine the effectiveness and efficiency of the proposed IDSs and compare them against various machine learning baseline models for identifying and categorizing malware that uses clock-gating.

The remaining part of this paper follows this structured outline: In Section 2, we review prior research pertaining to the topic at hand. Section 3 outlines the methodology applied in this study, while Section 4 presents the analysis and findings. Finally, in Section 5, we conclude and discuss potential future lines of exploration.

## 2. Related Work

This section aims to highlight the emergence of malware threats to clock-gating operation and refine malware identification and classification within the embedded systems landscape. Previous research works have explained and analyzed clock-gating and other ways of preventing power dissipation. In addition, threats to the hardware of embedded systems have been analyzed. However, little research has been done on threats to clock-gating and the software involved in embedded systems. For these reasons, we seek ways to improve and rectify these shortcomings. We want to identify and classify malware in embedded systems correctly.

Several works have been proposed to secure embedded systems [9,10,11]. Zareen et al. [8] present an approach to embedded device security through the development of a Hardware Immune System (HWIS), leveraging Artificial Immune Systems for effective malware detection in IoT devices. In resource-constrained environments, the HWIS demonstrates high efficiency in detecting botnet activities, achieving 96.7% accuracy with minimal overhead in power and area and no impact on processor delay. In the context of IoT device security, this method represents a significant improvement over traditional software-based malware detection.

Tamil et al. [12] described the use of clock-gating, which decreases the dissipation of dynamic power in synchronous circuits. The paper explained how clock-gating works and also outlined different clock-gating techniques. Various types were considered, such as latch-based, flip-flop-based, gate-based, synthesis-based, and look-ahead-based clock-gating. The paper also discussed other power reduction techniques, such as power gating and adiabatic logic. In conclusion, the paper presented a summary of the issues that are associated with clock-gating.

Shila et al. [13] proposed the design and implementation of Hardware Trojan Threats (HTTs) in Field-Programmable Gate Arrays (FPGA). The paper also proposed a detectability metric, called HTT detectability metric (HDM), to assess the efficiency of HTT detection techniques. A security analysis of the HTTs was conducted, and their detectability was evaluated using the proposed metric. Testbeds were put into use on MicroZed’s Xilinx Zynq-based FPGA development board. The paper showed that the proposed HTTs can be successfully implemented in the FPGA testbed. The detectability metric proposed by the paper effectively evaluated the detectability of HTTs. The security analysis of the HTTs showed that they can be used to leak secret information or cause denial-of-service attacks.

Subramanian et al. [14] proposed an Adaptive Counter-Clock (ACC) S-Box algorithm for Advanced Encryption Standard (AES) [15] that corrects errors while encryption takes place, as well as ensuring the security of data during encryption. The paper also aimed to reduce area size, power dissipation, and consumption. The round keys were obtained by running a key expansion code on three different key lengths of AES (128, 192, and 256 bits). Errors in data encryption were fixed using the ACC S-Box technique. As part of the encryption process, the paper made use of Field-Programmable Gate Arrays (FPGAs). The results show that the ACC S-Box algorithm improves the security of AES by rectifying errors during data encryption.

Mehta et al. [16] have proposed a method for detecting suspicious activity in Internet of Things (IoT)-embedded devices. The proposed method is based on a hierarchical design that distributes computational resources over IoT devices, making it scalable. The approach observes the device’s performance and correlation to similar devices to detect anomalies. Experiment findings demonstrate that the proposed strategy effectively identifies suspicious activity. The proposed approach is also resilient, meaning it can continue operating with minimum functionality even if an intrusion is detected.

Hunter et al. [17] investigated the viability of resource-constrained embedded devices frequently utilized in Internet of Things (IoT) systems by utilizing deep learning for intrusion detection. In the paper, four deep learning models that had already been trained were tested on devices with different capacities for resources. The models were trained on separate intrusion detection datasets, and their accuracy, precision, recall, F1 score, and prediction rate were evaluated. The paper also included testing the models’ responses to new attack patterns using separate datasets. The research also covered the usage of thin neural network structures for outstanding performance with little computation and potential consumption of energy. The study’s findings, which assessed whether deep learning-based intrusion detection could be implemented on embedded devices with minimal resources, were given in the publication. According to the paper’s findings, lightweight neural network topologies can deliver enough performance with few calculations and potential power requirements.

Emnett et al. [18] discuss a design methodology using RTL clock-gating in ASICs to significantly reduce power consumption, with a successful application in a 200K-gate ASIC reducing power by two-thirds. The method also integrates with full scan techniques for low-power and testable designs.

Shinde et al. [19] investigate various clock-gating techniques for power optimization in VLSI circuits at RTL level, used extensively in the Pentium 4 processor. The paper emphasizes the importance of considering power optimization early in the design process, at the RTL stage.

Wu et al. [20] propose two clock-gating techniques based on a quaternary variable model of the clock in sequential circuits. The method demonstrates power savings and the potential for synchronous operation with the master clock, while also addressing engineering challenges for practical application.

Li et al. [21] introduce deterministic clock-gating (DCG) for microprocessors, showing an average of 19.9% reduction in processor power with no performance loss. DCG is contrasted with pipeline balancing (PLB), demonstrating greater power savings and simpler implementation.

Casillo et al. [22] present an embedded Intrusion Detection System (IDS) for automotive cybersecurity, using a Bayesian Network approach to quickly identify malicious messages in the vehicle’s Controller Area Network (CAN-Bus). Initial experiments with an automotive simulator show promising results for the system’s effectiveness.

Sayadi et al. [23] propose a lightweight, machine learning-based HMD framework for embedded devices, utilizing Hardware Performance Counter (HPC) features for runtime malware detection. The research highlights that while complex classifiers like MLP, BayesNet, and SMO show higher detection accuracy, lightweight classifiers like JRip and OneR offer high accuracy per unit area for different malware classes. The study demonstrates a significant improvement in malware detection accuracy using the customized HMD approach, providing insights into selecting suitable ML classifiers for embedded system malware detection.

Rahmatian et al. [24] present a hardware-assisted intrusion detection technique for secure embedded systems, focusing on real-time detection of malware execution. The method uses FPGA logic to detect behavioral differences between correct system operation and malware and is adaptable to new malware and changing system behaviors. The system extracts the Process ID (PID) from the OS, using it to monitor system call sequences on the FPGA. The technique is shown to be effective in handling real-world programs with minimal runtime performance overhead, making it a promising approach for application-specific embedded processors requiring fast and accurate attack detection.

Previous research underscores the evolving challenges in power optimization and malware detection in embedded systems, with a focus on clock-gating techniques and hardware-assisted solutions. While these studies lay a solid groundwork, our research distinguishes itself by specifically addressing the vulnerabilities in ARM Cortex-M-based microcontrollers. We propose an innovative Intrusion Detection System (IDS) tailored for these systems, utilizing advanced machine learning techniques for heightened accuracy in detecting and categorizing clock-gating malware, a crucial step forward in bolstering the security of modern embedded platforms.

## 3. System Architecture and Attack Models

**Figure 1:**
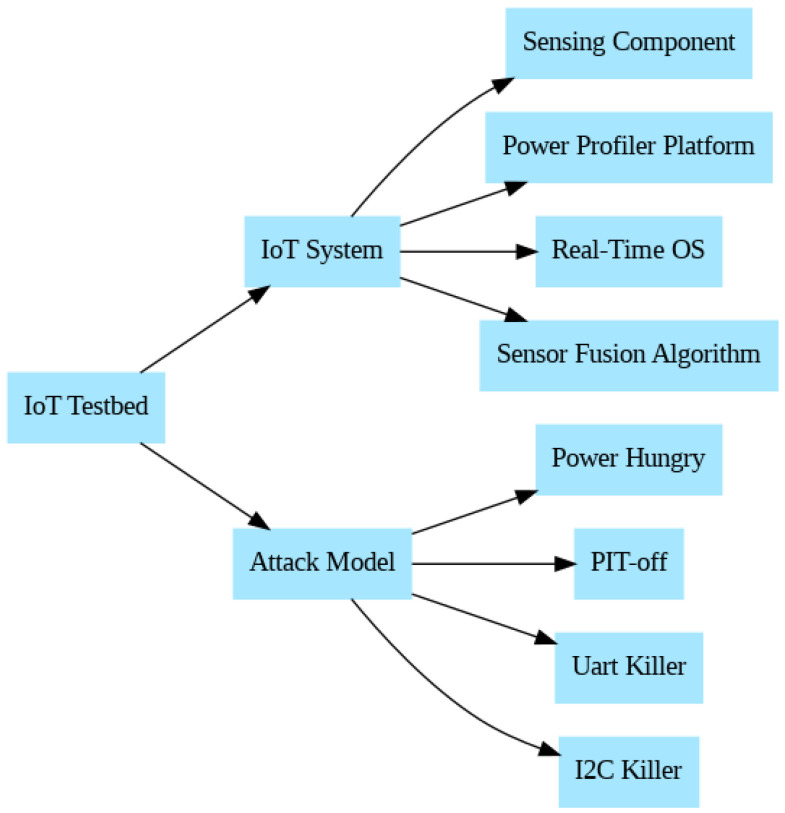
System architecture and attack models.

In this section, we describe the malware types that were implemented, how they were achieved, and the effect of the malware. In addition, we show the details of the testbed for the experiments. Figure 1 illustrates the components of the IoT malware testbed system and the four types of malware deployed.

### 3.1. System Architecture

The testbed consists of the experiments’ hardware and software development boards. The hardware components include the IoT system, sensing components, and power profiler platform. The software components consist of a real-time embedded operating system and a sensor fusion algorithm.

#### 3.1.1. Hardware Component

- IoT System: To evaluate the proposed malware code, a Freedom-K64F [25] low-cost development board was used. Two free software operating systems that facilitate IoT implementation on the board are ARM mbed OS version 5.0 and Zephyr OS version 3.5.0. The ARM Cortex-m4 processor (ARM, Cambridge, UK) has (MK64FN1M0VLL12 MCU) 256 KB of RAM, 1 MB of flash memory, and 120 MHz clock rate; the board features dual-role USB connectors, Ethernet, and SDHC [26,27].
- Sensing Component: As part of the proposed IoT testbed, a sensor board, namely, the FRDM-STBC-AGM01 is included. There is a three-axis motion sensor on the sensor shield (with a selectable sensitivity of ±2 g/±4 g/±8 g) and a 3D magnetometer. Its purpose is to facilitate the testing of various malware types within a comprehensive IoT system environment.
- Power Profiler Platform: Nordic Semiconductor has developed the Power Profiler Kit II to measure the power usage of infected IoT systems. This platform has a broad dynamic span from 1 µA to 1 A with a resolution ranging from 100 nA to 1 mA and a sampling rate of 100k samples each second. Power spectral density data were gathered to examine the proposed malware’s behavior.

**Figure 2:**
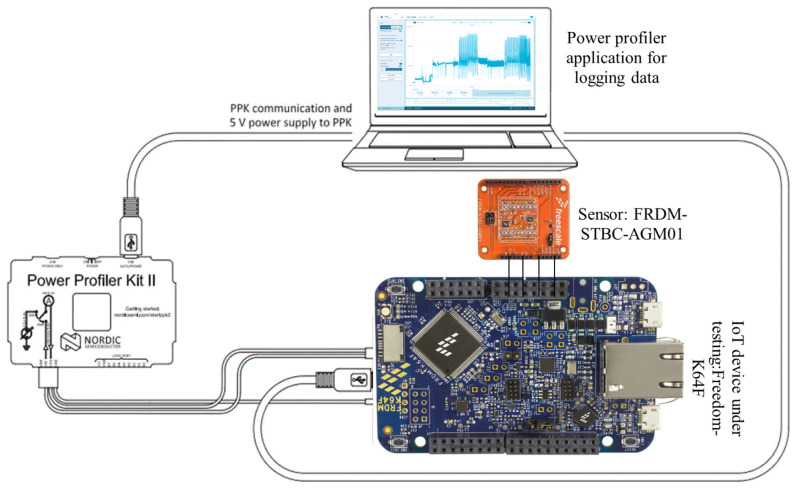
Hardware component.

Figure 2 provides a visual representation of these hardware components, showcasing their configuration and interconnectivity within the testbed.

#### 3.1.2. Software Component

- Real-time Embedded Operating System: On the testbed, an ARM Mbed 5.0 OS (ARM, Cambridge, UK) is used. Mbed OS is capable of running multithreaded IoT programs in real time for rapid prototyping. To implement the malware code, the file system_MK64F12.c within the Mbed OS was modified; the “systemInit()” function was modified as well [28]. In Mbed OS, the code for modifying the contents of control registers controlling the clock-gating of each malware type is contained. A code compilation and flashing of the OS code were then performed on the IoT testbed.
- Sensor Fusion Algorithm: For the FRDM-STBC-AGM01 sensor to measure the impact of real-time malware operating on the IoT system, software code is required. To implement and deploy the sensing fusion algorithm on the IoT system, an Mbed online compiler tool was used.

### 3.2. Attack Model

On the testbed, there are four types of malware deployed. The malware types include Power Hungry, PIT-off, uart killer, and $I^{2}C$ killer.

#### 3.2.1. Power Hungry

**Figure 3:**
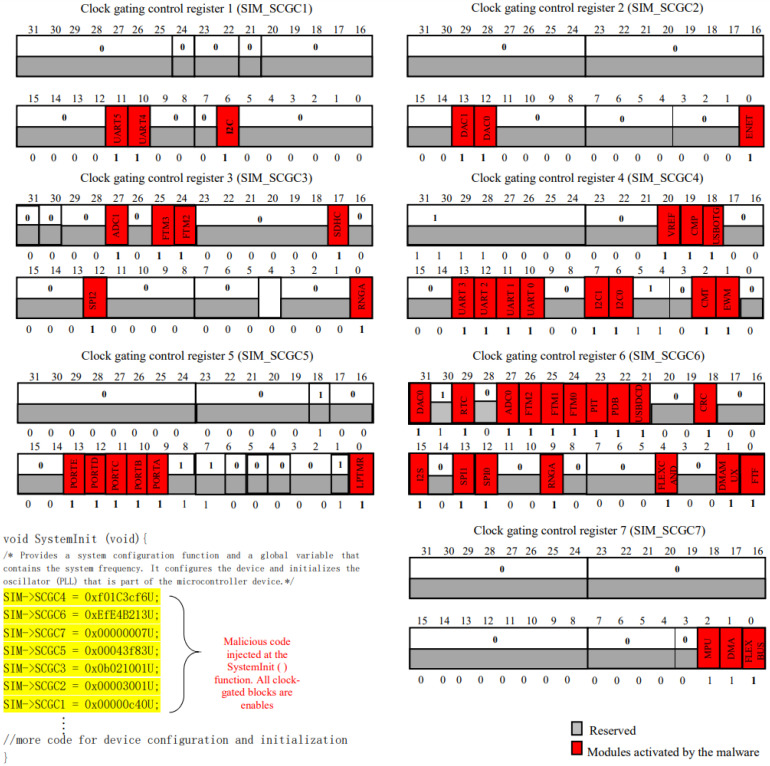
Code sample for Power Hungry malware utilizing SIM-SCGC* register contents (Rasheed et al. (2021) [1]).

This form of malware allows unauthorized access to the chip’s clock signals during system startup, causing excessive energy consumption and rapid battery drain. Its primary aim is to disrupt the system’s functionality by keeping all chip modules active, irrespective of their power states (e.g., Run, Wait, Stop). In the example provided (see Figure 3), the malware manipulates clock-gating control registers by setting their bits to high values. The SIM module is utilized to change these register values, specifically the SIM_SCGC1 gating control variable. This manipulation involves injecting code, such as “SIM->SCGC1=0x0000c40U”, into the “systemInit()” method. As a consequence of this action, it activates the uart4, aurt5, and $I^{2}C$ modules, while setting their clock-gate control bits to 1.

#### 3.2.2. PIT-Off

**Figure 4:**
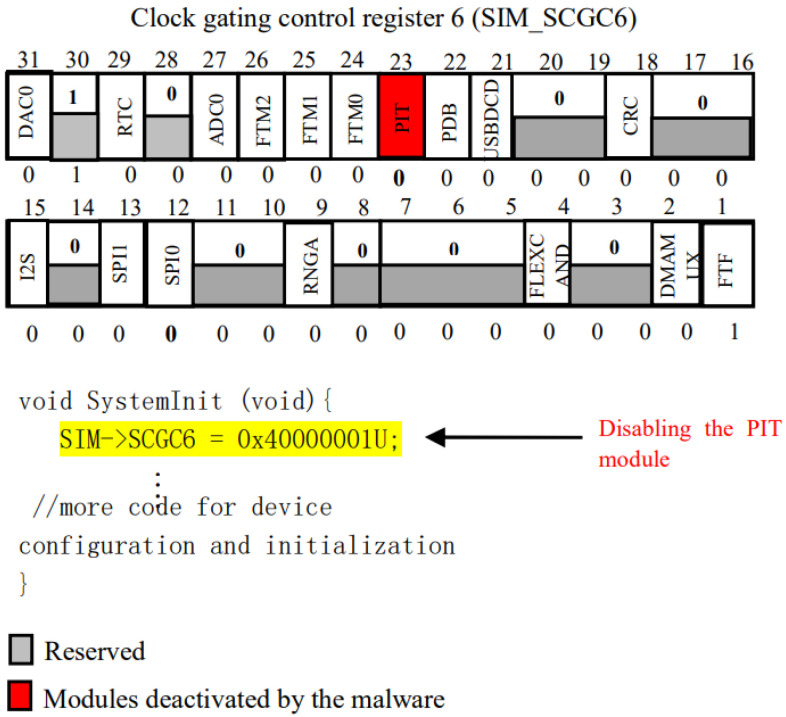
Code example for disabling PIT-off malware using the contents of SIM-SCGC6 registers (Rasheed et al. (2021) [1]).

The Periodic Interrupt Timer (PIT) module plays a crucial role in generating timed interrupts within a system. However, a specific type of malware can disrupt the PIT module, leading to the blockage of external hardware that relies on serial communication. Through embedding malicious code into the “systemInit()” method via dynamically online mbed OS changes, this malware successfully achieves its goal of deactivating the PIT module. As a consequence, this disruption causes runtime errors when external sensor modules attempt to transmit information to the IoT system. Consequently, the IoT system enters a perpetual boot cycle, rendering it nonfunctional. An illustrative example of this malware’s code insertion can be seen in Figure 4, where code such as SIM-> SCGC6 = 0x40000001U is employed to access and manipulate the PIT’s bit module content during the “systemInit()” method.

#### 3.2.3. Uart Killer

**Figure 5:**
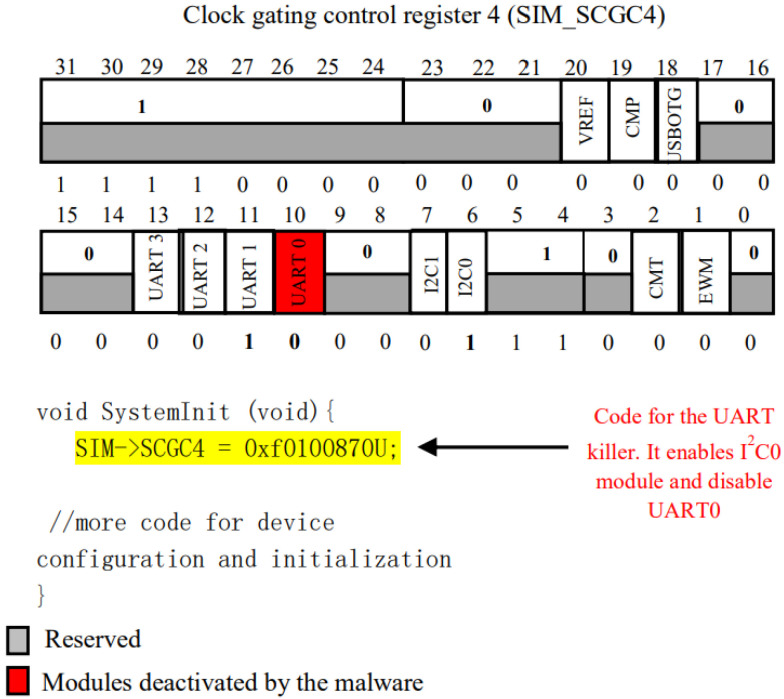
Code Example for uart killer malware leveraging SIM-SCGC4 register contents (Rasheed et al. (2021) [1]).

Embedded systems affected by the strain of the power-off-uart malware experience a disruption in their clock-gate signals associated with uart modules, resulting in the disabling of these modules. Consequently, peripheral devices and sensor components lose their ability to communicate data with the processor through the uart bus. This situation may result in the risk of data loss. The mechanics of the uart malware attack are visually depicted in Figure 5.

#### 3.2.4. $I^{2}C$ Killer

**Figure 6:**
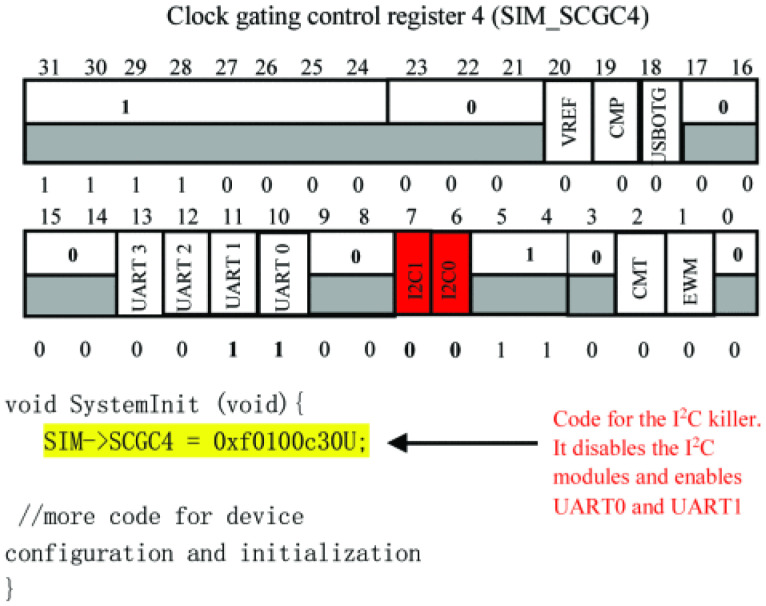
Code sample for $I^{2}C$ killer malware utilizing SIM-SCGC4 register contents (Rasheed et al. (2021) [1]).

The $I^{2}C$ killer behaves similarly to the uart killer by disabling serial communication modules. In contrast to the uart killer, it deactivates the $I^{2}C$ module while configuring the clock signals for the uart0 and uart1 modules. In SIM->SCGC4 = 0xf0100c30U, b7 and b6 are cleared to 0, while b10 and b11 are set to 1 in the SCGC4 register. Figure 6 illustrates the $I^{2}C$ malware attack.

## 4. Proposed Methodology

**Figure 7:**
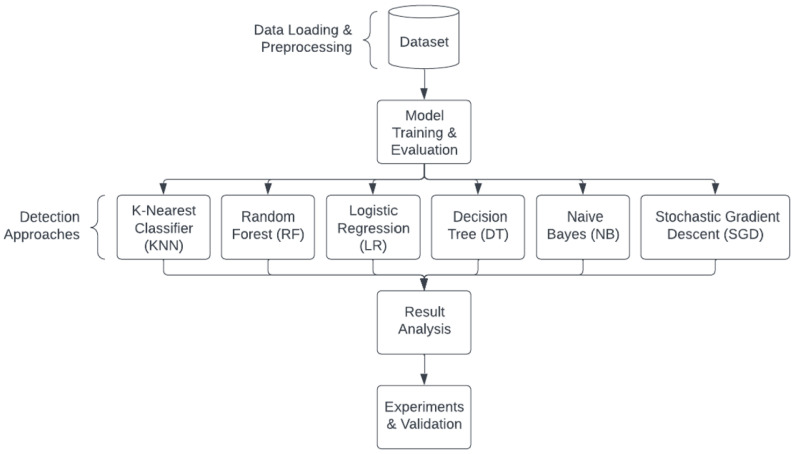
The methodology of the proposed Intrusion Detection System (IDS) for malware detection on embedded systems.

Figure 7 illustrates the methodological steps employed in this research to develop and evaluate an Intrusion Detection System (IDS) for detecting and classifying malware on embedded systems. According to Figure 7, here are our methodology steps:

- Data Loading and Preprocessing: This initial step involves loading the dataset used for experimentation and preprocessing the data. The dataset consists of two features, time and current, and is labeled with distinct classes representing different malware types.
- Model Training and Evaluation: The preprocessed dataset is used to train the chosen machine learning models. This involves feeding the models with labeled data and allowing them to learn patterns and features associated with malware detection. Subsequently, the trained models are evaluated using appropriate metrics to assess their performance.
- Machine Learning Models: In this phase, various traditional machine learning models are considered for the IDS. These models include K-Nearest Classifier (KNN), Random Forest (RF), and Logistic Regression (LR).
- Result Analysis: The outcome of model training and evaluation is thoroughly analyzed. Evaluation metrics such as accuracy scores, as mentioned in the paper, are used to measure the effectiveness of the IDS in detecting and classifying malware.
- Experiments and Validation: The authors conduct experiments to compare the performance of different machine learning models.

The algorithm used to carry out the experiment is described in Algorithm 1.
**Algorithm 1** Classification Algorithm.  1:*D*← LoadData(); where $D=[d_{1},d_{2},d_{3},\ldots ,d_{n}]$        ▹ Load data into dataset  2:Check if $d_{k}$∈*D* is empty or null; impute if yes  3:Initialize *D* as feature vector: $X=[x_{1},x_{2},x_{3},\ldots ,x_{n}]$  4:Normalize feature vector: X=normalize(X)  5:Split feature vector *X* into training ($X_{\mathrm{train}}$) and testing ($X_{\mathrm{test}}$) data  6:model_list=[Model1,Model2,Model3,…,Model6]  7:model_prediction =[0]×len(original_list)      ▹ List to hold model predictions  8:iterations=10  9:total_prediction=010:**for** model in model_list **do**11:    **for** 1:iterations **do**12:        Initialize model13:        Train $model(X_{\mathrm{train}})$14:        prediction = Test $model(X_{\mathrm{test}})$15:        total_prediction+=prediction16:    **end for**17:    model_prediction[model]=total_prediction/Iterations18:    print classification report19:    total_Prediction=020:**end for**

### 4.1. Dataset

This study involved the collection of current/power consumption data under normal device operation and when the device was infected. Various malicious codes, including Power Hungry, PIT-off, $I^{2}C$ killer, and uart killer, were executed on separate IoT platforms, simulating a total duration of 600 s. Current/power measurements were recorded at a sample rate of 1000 samples per second, with a current resolution of 1 µA. For each malware strain, 600,000 data points were gathered during the experiment. Additionally, a dataset comprising 700,000 current measurements was collected for an IoT testbed without infection.

### 4.2. Intrusion Detection System Based on Machine Learning Approaches

The proposed Intrusion Detection System (IDS) is designed to detect malware on embedded systems based on the “systemInit()” function. The “systemInit()” method is responsible for initializing the system after booting. We intend to check the “systemInit()” during boot time to detect and correctly classify malware types. The IDS uses the signature-based technique for malware detection. The signature-based detection technique detects known malware based on its signature and pattern.

The design consists of two main components: preprocessing and detection modules. The preprocessing module is responsible for collecting and preprocessing data from the “systemInit()” method. This module collects the necessary data that are needed to detect malware. The collected data are then preprocessed to extract features. The detection module uses machine learning models to classify the extracted features as malicious or benign. It will also classify the subcategory of the malware where necessary.

Our proposed IDS is expected to be an effective tool for detecting malware on embedded systems. By monitoring the “systemInit()” method and using machine learning to classify the extracted features, the IDS can detect and respond to malware in real time, preventing potential damage to the system. We have employed various traditional machine learning models to detect and classify clock-gating-assisted malware. Six machine learning approaches were utilized for the proposed IDS, namely, K-Nearest Classifier, Random Forest, Logistic Regression, Decision Tree, Naive Bayes, and Stochastic Gradient Descent.

#### 4.2.1. K-Nearest Classifier (KNN)-Based Detection Approach

This is a classification algorithm that is commonly used in machine learning. To implement the K-Nearest Classifier algorithm, we used Python and its built-in library for machine learning, sci-kit-learn. To optimize the algorithm’s performance, we experimented with different values of the hyperparameter k, which determines the number of nearest neighbors to consider when classifying a new sample. Tuning this hyperparameter improved classification accuracy, especially for the clock-gating-assisted malware dataset [29].

The K-Nearest Neighbors classification formula is a fundamental concept in machine learning for classifying data points based on the majority class of their nearest neighbors. $\hat{Y}\left(x\right)$ represents the data point’s expected class label *x*. $\arg\max_{j}$ is used to find the class label *j* that maximizes a specific expression. $\sum_{i=1}^{k}$ represents the summation over *k* terms, where *k* is the number of nearest neighbors considered. $I(y_{i}=j)$ is an indicator function that equals 1 when $y_{i}=j$, indicating that the *i*th neighbor belongs to class *j*.

$$\hat{Y}\left(x\right)=\arg\max_{j}\sum_{i=1}^{k}I\left(y_{i}=j\right)$$

#### 4.2.2. Random Forest (RF)-Based Detection Approach

Multi-decision tree ensemble learning improves classification accuracy by combining multiple decision trees. With Python and scikit-learn, we implemented the Random Forest algorithm and tuned its hyperparameters, including the number of trees and their maximum depth. By using this algorithm, we were able to achieve high accuracy in detecting and classifying clock-gating-assisted malware. Although Random Forests are robust and perform well on many datasets, they can be computationally expensive and may overfit on noisy datasets [30]. In the formula below, $\hat{Y}\left(x\right)$ represents the predicted class label for the data point *x*. It is determined by taking the mode (the most frequently occurring class label) of the predicted class labels from *n* individual decision trees, where $Y_{i}\left(x\right)$ is the prediction made by the *i*-th decision tree. Random Forest leverages the diversity of multiple trees to improve the overall accuracy and generalization of the classification, making it a powerful machine learning algorithm for various tasks.

$$\hat{Y}\left(x\right)=\mathrm{mode}\left\{Y_{1}\left(x\right),Y_{2}\left(x\right),\ldots ,Y_{n}\left(x\right)\right\}$$

#### 4.2.3. Logistic Regression (LR)-Based Detection Approach

For binary classification applications, the logistic regression approach is frequently utilized. We implemented the Logistic Regression algorithm using Python and scikit-learn, and we tuned the hyperparameters, including the regularization strength and the solver used. By fine-tuning these hyperparameters, we were able to improve the accuracy of the algorithm in detecting and classifying clock-gating-assisted malware. We were able to improve the accuracy of the algorithm in detecting and classifying clock-gating-assisted malware. Logistic Regression is simple and efficient but may not perform well when the decision boundary is nonlinear [31]. In the formula below, P(Y=1|X) represents the conditional probability that the target variable *Y* takes the value 1 given the input features *X*. The equation involves model parameters $w_{0},w_{1},w_{2},\ldots ,w_{n}$ that are learned during training, and $x_{1},x_{2},\ldots ,x_{n}$ are the input feature values. The logistic function $\frac{1}{1+e^{-z}}$, where *z* is the linear combination of the features and parameters, is used to model the probability of the positive class. One-vs.-all (OvA) or softmax regression approaches can be used to expand the utility of logistic regression to multiclass issues, which are particularly beneficial for binary classification applications.

$$P\left(Y=1|X\right)=\frac{1}{1+e^{-(w_{0}+w_{1}x_{1}+w_{2}x_{2}+\ldots +w_{n}x_{n})}}$$

#### 4.2.4. Decision Tree (DT)

This is a widely used algorithm in machine learning that is commonly used for classification tasks. Based on a tree-like model, decisions are modeled along with their possible consequences. Using a recursive process, the algorithm divides the data into smaller groups at every tree node by focusing on the most important feature. The Decision Tree algorithm’s hyperparameters, such as the tree’s deepest point and the lowest possible number of samples necessary to divide a node, were adjusted using grid search. The Decision Tree is easy to interpret and can handle categorical and numerical data, but it can easily overfit and perform poorly on complex datasets [32]. A Decision Tree, denoted as *T*, recursively partitions the feature space into regions by evaluating feature conditions at each node. *T* represent a Decision Tree with nodes $N_{i}$, feature conditions $F_{i}$, and child nodes $N_{iL}$ and $N_{iR}$:

$$T\left(N_{i}\right)=\left\{\begin{array}{c} \mathrm{Leaf}\;\mathrm{node},\;\mathrm{if}\;N_{i}\;\mathrm{is}\;a\;\mathrm{terminal}\;\mathrm{node}\\ T\left(N_{iL}\right),\;\mathrm{if}\;F_{i}\left(x\right)=\mathrm{True}\;\mathrm{for}\;x\;\mathrm{and}\;N_{i}\;\mathrm{is}\;\mathrm{not}\;a\;\mathrm{terminal}\;\mathrm{node}\\ T\left(N_{iR}\right),\;\mathrm{if}\;F_{i}\left(x\right)=\mathrm{False}\;\mathrm{for}\;x\;\mathrm{and}\;N_{i}\;\mathrm{is}\;\mathrm{not}\;a\;\mathrm{terminal}\;\mathrm{node} \end{array}\right.$$

#### 4.2.5. Naive Bayes (NB)

This is a probabilistic method that is frequently used for classification tasks, particularly in Natural Language Processing (NLP). We implemented the Naive Bayes algorithm using the scikit-learn library in Python. We tuned the hyperparameter alpha, which controls the strength of the smoothing applied to the probabilities. To improve the accuracy of the algorithm, we experimented with different values of the hyperparameter “alpha”, which controls the strength of the smoothing applied to the probabilities. Naive Bayes is fast and efficient for high-dimensional datasets, but it assumes independence between features and may perform poorly when this assumption is violated [33]. Naive Bayes is a probabilistic classifier that estimates the probability of a data point belonging to a particular class *y* based on the likelihood of the features *X* given that class and the prior probability of class *y*. It simplifies the computation by assuming that characteristics are conditionally independent. In the following equation, P(Y=y|X) signifies the probability that a particular class *y* is the correct one given the input features *X*. P(X|Y=y) represents the likelihood of encountering the input features *X* when the class is *y*. P(Y=y) indicates the initial probability of class *y*, while P(X) refers to the overall probability of observing the input features *X*.

$$P\left(Y=y|X\right)=\frac{P(X|Y=y)\cdot P(Y=y)}{P(X)}$$

#### 4.2.6. Stochastic Gradient Descent (SGD)

An iterative optimization algorithm is used to train large-scale machine learning models. At each iteration, the model’s parameters are updated by computing the gradient of the loss function on a subset of the training data. It can optimize logistic regression or linear SVM models for classification tasks. Hyperparameters such as learning rate, regularization parameter, and batch size can be selected using grid search and cross-validation. Due to its efficiency and effectiveness in large-scale problems, SGD is widely used in machine learning libraries [34]. In the formula below, $w_{t+1}$ represents the updated model parameters at iteration t+1, $w_{t}$ is the current model parameter vector at iteration *t*, η is the learning rate that controls the step size, and $\nabla L(w_{t})$ is the gradient of the loss function $L(w_{t})$ with respect to the model parameters. SGD is suitable for large datasets and online learning since it iteratively changes the model variables in a direction that minimizes the loss. The learning rate η plays a crucial role in controlling the step size and convergence speed.

$$w_{t+1}=w_{t}-\eta \nabla L\left(w_{t}\right)$$

## 5. Performance Analysis

### 5.1. Experimental Setup

Configuring the hardware and software environments is crucial for strong and repeatable experiments. This section details the carefully selected settings that supported our research, ensuring the reliability and scalability of our research.

#### 5.1.1. Hardware Configuration

In this study, Google Colab, a cloud-based platform known for its versatility in facilitating machine learning experiments, was used to facilitate the computation. The study’s computational infrastructure was enhanced by utilizing Google Colab’s “Pro” subscription, which provided access to premium resources. To speed up model development and training, both A100 and V100 Tensor Core GPUs (NVIDIA, Santa Clara, USA) were utilized within this subscription.

The availability of premium GPUs played a pivotal role in the research process. For computing-intensive experiments, the V100 GPUs, distinguished by their exceptional computing capabilities, were strategically employed. The A100 GPUs provided robust performance for various machine learning tasks. As a result of this dynamic allocation of GPU resources, machine learning models were trained efficiently across 100 epochs of experiments.

#### 5.1.2. Software Configuration

Using Google Colab, the software environment was meticulously configured to integrate hardware resources and essential software tools. The Google Colab environment accommodated a wide range of software components:

- Operating System: The research was conducted within the Google Colab environment, eliminating the need to manage the operating system manually. By abstracting the underlying operating system complexity, Colab provided a consistent and reliable environment.
- Python: For modeling, Python served as the foundational programming language. It is regarded as one of the most prominent languages in machine learning. The codebase was executed using Python 3.10, enabling access to various machine learning libraries and frameworks.
- Machine Learning Libraries: The study leveraged an ensemble of machine learning libraries, including Scikit-learn, Keras, and TensorFlow. The Colab environment makes it easy to develop and evaluate machine learning models using these libraries.
- Data Preprocessing Tools: With Scikit-learn’s robust preprocessing module, data preprocessing tasks such as data cleaning, feature scaling, and encoding were seamlessly performed.
- Hyperparameter: For each model, we use a different set of parameters and hyperparameters. We use the Gini impurity as the criterion to partition at each node and set the maximum depth to three in the Decision Tree classifier. Both parameters were chosen due to their simplicity computational simplicity. The Gini impurity is also suitable for multiclass classification. The maximum depth was also set to three because it gave the best result during testing. Similarly, in the Random Forest model, the random state is set at zero with a maximum depth of three. In KNN, the number of neighbors is set to seven. To determine the K-value, the odd values were first tested since this eliminates ties and gives a majority class. As a result, we tested K values of 3, 5, and 7, with 7 showing the highest level of performance. For SGD, the maximum iteration parameter is 100. The value was chosen based on resource availability and faster convergence of the SGD model. In conclusion, we used different values and combinations of parameters and hyperparameters to achieve the best results.

### 5.2. Metrics

A set of key performance metrics was used to evaluate our models, including precision, recall, accuracy, and F1-score. Each of these metrics is crucial in assessing the model’s performance.

The accuracy of the model’s favorable predictions is referred to as precision. It indicates the proportion of accurate positive predictions out of all positive examples. A model’s precision measures its ability to identify relevant instances while minimizing false positives. Precision is defined as: $\mathrm{Precision}=\frac{\mathrm{True}\;\mathrm{Positives}}{\mathrm{True}\;\mathrm{Positives}+\mathrm{False}\;\mathrm{Positives}}$.

A model’s recall measures how well it can identify all relevant instances. It reflects the percentage of real positive predictions that were successfully detected, based on all actual positive events. The majority of positive cases are accurately captured by a model with a high recall. Recall is defined as $\mathrm{Recall}=\frac{\mathrm{True}\;\mathrm{Positives}}{\mathrm{True}\;\mathrm{Positives}+\mathrm{False}\;\mathrm{Negatives}}$.

A model’s accuracy is measured by how well it predicts the future. From all instances in the dataset, it represents the percentage of correctly classified instances (both true positives and true negatives). For all classes, accuracy provides an overview of the model’s performance. It is defined as $\mathrm{Accuracy}=\frac{\mathrm{True}\;\mathrm{Positives}+\mathrm{True}\;\mathrm{Negatives}}{\mathrm{True}\;\mathrm{Positives}+\mathrm{True}\;\mathrm{Negatives}+\mathrm{False}\;\mathrm{Positives}+\mathrm{False}\;\mathrm{Negatives}}$.

An F1-score is a harmonic mean of precision and recall. This metric measures the model’s ability to achieve high precision and recall simultaneously. In imbalanced datasets, where precision and recall may trade off, the F1-score is especially useful. It is defined as $\mathrm{F1-score}=2\times \frac{\mathrm{Precision}\times \mathrm{Recall}}{\mathrm{Precision}+\mathrm{Recall}}$.

Ultimately, these performance metrics provide a nuanced evaluation of machine learning models, encompassing precision, recall, accuracy, and F1-score. By considering these metrics, we can determine how well the models perform in various aspects of classification and prediction.

### 5.3. Results and Discussion

In this section, we delve into the findings of our study, starting with the visualization of our data and extending through the performance of various machine learning models in classifying clock-gating-assisted malware. Our results show the effectiveness of our approach as well as the complexity of the task.

#### 5.3.1. Initial Data Characterization

**Figure 8:**
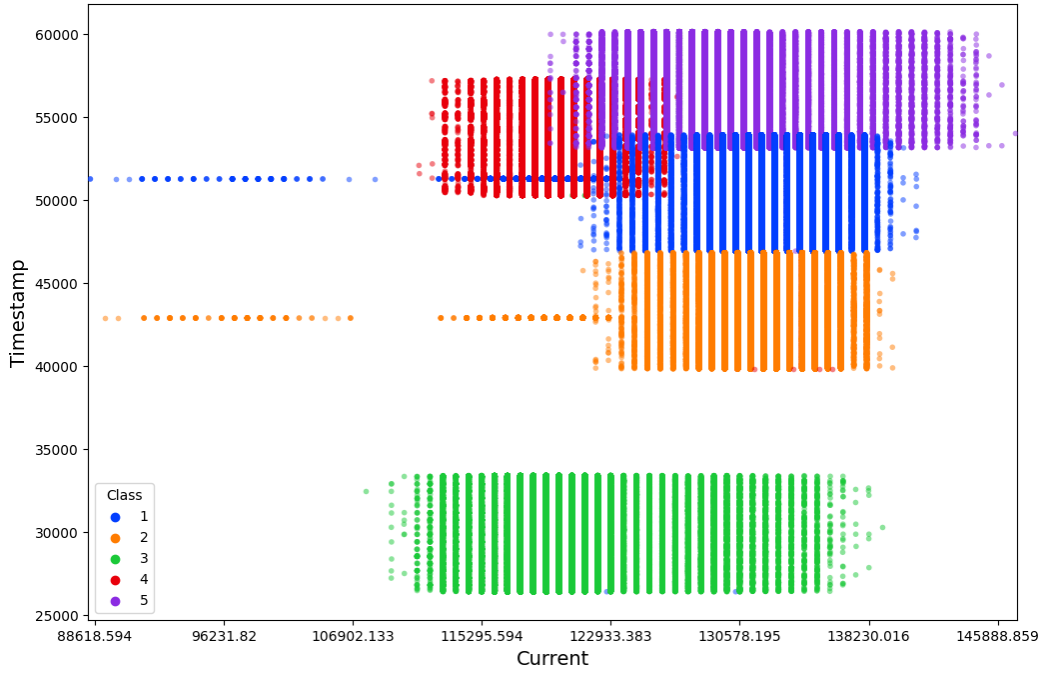
Scatter plot of the malware types and normal operations.

Before applying machine learning algorithms, it is critical to understand the data’s inherent structure and any observable patterns. To this end, we employed a scatterplot, as depicted in Figure 8, to visualize the distribution of different malware types alongside normal operation data. Classes 1, 2, 3, 4, and 5 represent Power Hungry, $I^{2}C$ killer, normal operations, PIT-off, and uart killer, respectively. This preliminary analysis helps to set expectations regarding the complexity of the classification task and to underscore the necessity for sophisticated analytical techniques such as machine learning. It becomes evident from this visualization that while certain malware classes, specifically classes 1 and 2, appear similar in the context of current consumption, others are more distinctly separable, suggesting varied levels of difficulty that one might encounter during the classification process.

#### 5.3.2. Machine Learning Model Efficacy

Table 1 shows the comparison results of our proposed approaches. It can be seen that the K-Nearest Classifier and Logistic Regression achieved the highest accuracy, precision, recall, and F1-score values, above 0.97. The Decision Tree model achieved an accuracy of 0.80 and an F1-score of 0.73, slightly lower than the other models.

#### 5.3.3. Training Performance Analysis

**Figure 9:**
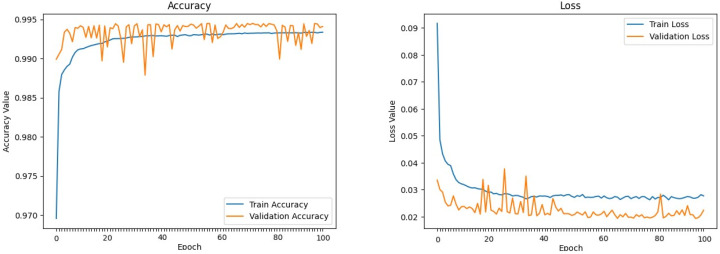
Decision Tree.

**Figure 10:**
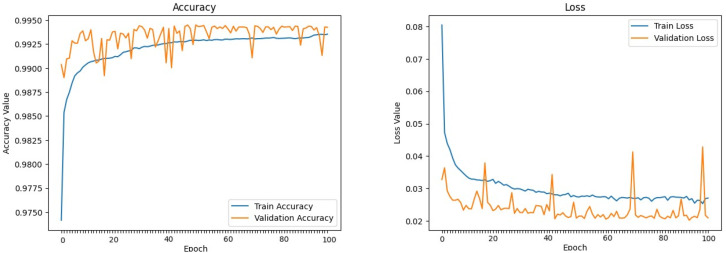
K-Nearest Neighbors.

**Figure 11:**
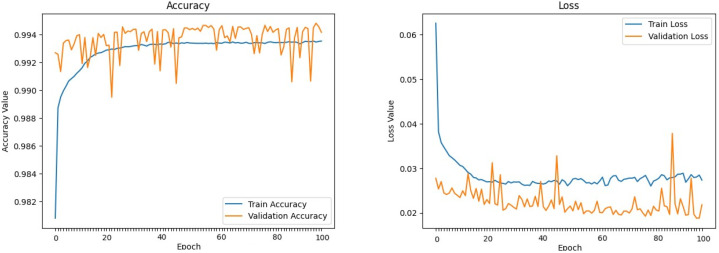
Linear Regression.

**Figure 12:**
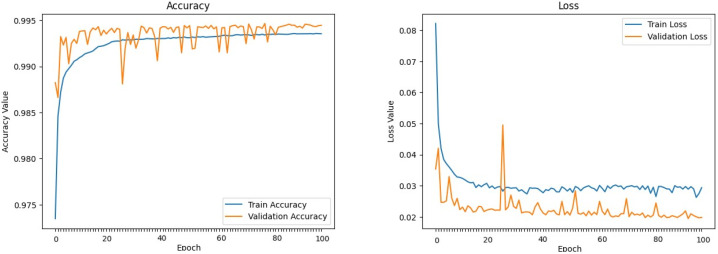
Naive Bayes.

**Figure 13:**
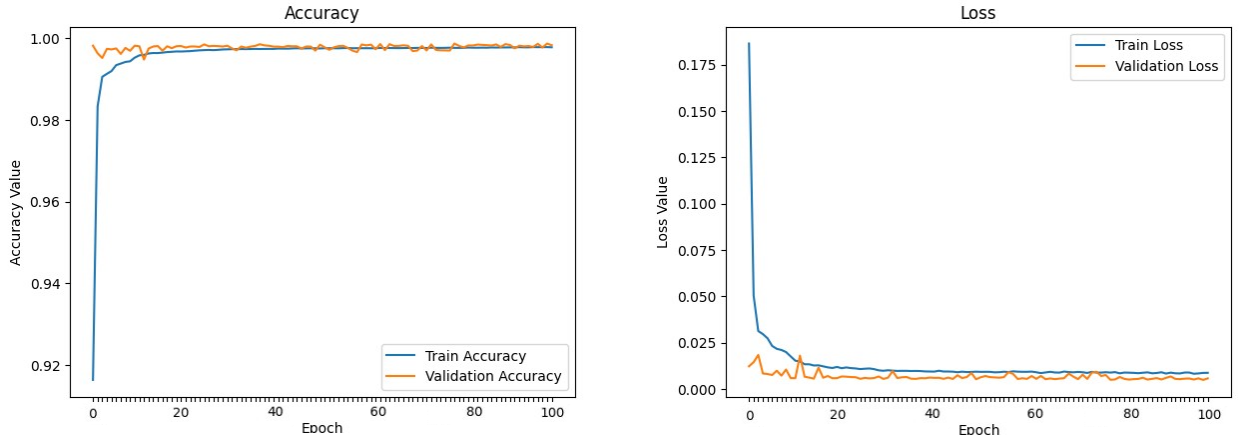
Random Forest.

**Figure 14:**
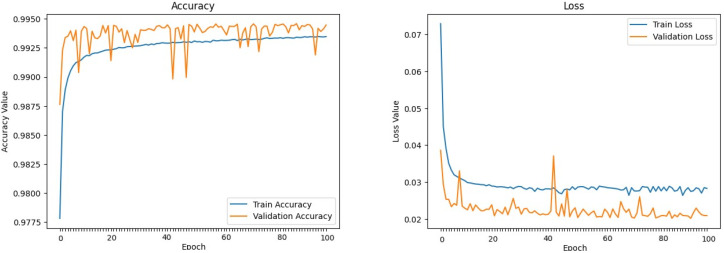
Stochastic Gradient Descent.

The machine learning models defined above were trained and evaluated over 100 epochs with our dataset, utilizing a data split of 70% for training, 15% for validation, and 15% for testing. Figure 9, Figure 10, Figure 11, Figure 12, Figure 13 and Figure 14 display accuracy and loss plots for both training and validation of Decision Tree, K-Nearest Neighbors, Linear Regression, Naive Bayes, Random Forests, and Stochastic Gradient Descents to evaluate their training performances. These figures provide an overview of each model’s training performance. The accuracy plots illustrate the models’ ability to learn from training data. Furthermore, the loss plots demonstrate the convergence of the models’ training processes, indicating their ability to minimize errors and improve prediction. Combined, these plots provide valuable insights into the training performances of the models and highlight their strengths and effectiveness.

#### 5.3.4. Post-Training Classification Insights

**Figure 15:**
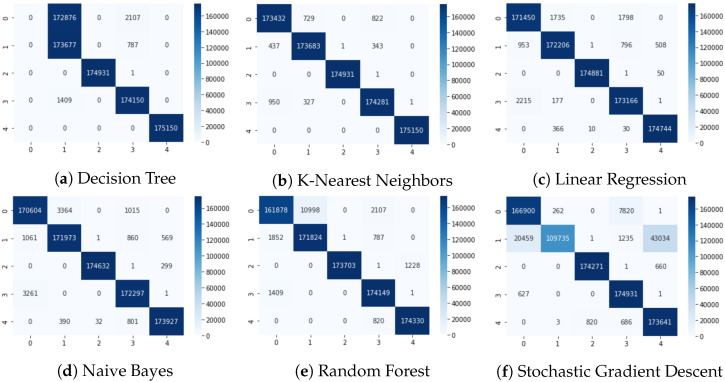
Confusion matrices for machine learning models.

After the training phase, we evaluated the performance of each model through confusion matrices, presented in Figure 15. The confusion matrices of machine learning models, including Decision Tree (a), K-Nearest Neighbors (b), Linear Regression (c), Naive Bayes (d), Random Forest (e), and Stochastic Gradient Descent (f) are shown. These matrices provide a stark contrast to the initial scatterplot Figure 8 by revealing the effectiveness of each algorithm in classifying the data post-learning. In these matrices, classes 0, 1, 2, 3, and 4 represent Power Hungry, $I^{2}C$ killer, normal operations, PIT-off, and uart killer, respectively.

The K-Nearest Neighbors (KNN) and Logistic Regression (LR) models notably outperformed other models with remarkably high accuracy rates of 99%. This impressive performance indicates not just their ability to learn from the training data but also their robustness in distinguishing between classes that appeared similar in the raw data. Their success in accurately classifying closely clustered data points, as seen in the scatter plot, validates their capability to handle real-world scenarios where malware types may not be distinctly separable.

The confusion matrices serve as a detailed record of each model’s classification strengths and potential areas for improvement. For instance, while the Decision Tree model demonstrated lower accuracy, this was mitigated by the Random Forest model, which leverages the power of multiple decision trees to improve the overall classification results. In light of this observation, careful model selection must be tailored to the specific characteristics of the dataset and the details of the classification task.

The efficacy of the KNN and LR models, in particular, suggests their strong potential for application in embedded systems for malware detection and prevention. These models have proven to be highly reliable in distinguishing clock-gating-assisted malware from legitimate operations. In contrast, the lower reliability of the Decision Tree model suggests that while it may contribute to an ensemble method like Random Forest, it might not be the best independent choice for this specific task.

In conclusion, the demonstrated ability of machine learning models, especially KNN and LR, to detect and prevent clock-gated malware holds great promise for enhancing the security framework of embedded systems. Their high performance in our evaluations underscores the valuable role that machine learning can play in improving system security and reliability against increasingly sophisticated cyber threats.

## 6. Conclusions

This study has presented a comprehensive approach to enhancing the security of embedded systems through the development of an Intrusion Detection System (IDS) that leverages machine learning techniques. By focusing on the classification of clock-gating-assisted malware, the research aimed to address the software threats that exploit clock-gating techniques in embedded systems. We identified and deployed four distinct types of malware on a testbed, namely, Power Hungry, PIT-off, uart killer, and $I^{2}C$ killer, to test the efficacy of the proposed IDS. The integration of machine learning models with the “systemInit()” method provided a real-time response capability, crucial for reducing potential damages to the system.

Based on the evaluation, we assessed the performance of several machine learning models: K-Nearest Classifier (KNN), Random Forest (RF), Logistic Regression (LR), Decision Tree (DT), Naive Bayes (NB), and Stochastic Gradient Descent (SGD). The models demonstrated a significant ability to detect and classify clock-gating-assisted malware, with accuracy scores ranging from 0.80 to 0.99. The KNN and LR models, in particular, showed exceptional performance and robustness, indicating their potential for real-world application in embedded systems security.

The findings suggest that machine learning models are not only capable of providing a reliable and efficient defense against clock-gating-assisted attacks but also show promise for the continued advancement of security measures in the face of sophisticated and evolving threats. Future research is encouraged to focus on refining these models to further enhance the detection and classification capabilities for a broader spectrum of malicious activities. Furthermore, the IDS could be extended to other types of embedded systems, adapting to different hardware configurations and operating environments. It may be possible to integrate real-time adaptive learning mechanisms in order to continually evolve in response to emerging malware threats in the future.

In conclusion, this paper underscores the critical need for implementing advanced security mechanisms in embedded systems. Embedding machine learning models into an IDS framework offers an effective way to protect embedded systems against the increasingly complex landscape of cyber threats.

## Figures and Tables

**Table 1:** Results for the machine learning models.

Model	Accuracy	Precision	Recall	F1-Score
Decision Tree	0.80	0.69	0.80	0.73
Stochastic G. D.	0.91	0.92	0.91	0.90
K-Nearest Classifier	0.99	0.99	0.99	0.99
Naive Bayes	0.98	0.98	0.98	0.98
Random Forest	0.97	0.97	0.97	0.97
Logistic Regression	0.99	0.99	0.99	0.99

## Data Availability

The dataset used during this project was generated from a dedicated testbed that is located in the Autonomous System and IoT Lab within Computer Science Department at Sam Houston State University. All generated datasets are privately stored in the department data center and our lab’s computing system. Datasets are made available to our research team to conduct various research experiments.

## References

[1] Rasheed A.A., Varol H., Baza M. Clock-gating-Assisted Malware (CGAM): Leveraging Clock-Gating on ARM Cortex M for Attacking Subsystems Availability. Proceedings of the 2021 9th International Symposium on Digital Forensics and Security (ISDFS).

[2] Ismail I., Nor S.M., Marsono M.N. (2014). Stateless Malware Packet Detection by Incorporating Naive Bayes with Known Malware Signatures. Appl. Comput. Intell. Soft Comput..

[3] Bace R., Mell P. (2001). Intrusion Detection Systems.

[4] Stavroulakis P., Stamp M. (2010). Handbook of Information and Communication Security.

[5] Rasheed A., Baza M., Khan M., Karpoor N., Varol C., Srivastava G. Using Authenticated Encryption for Securing Controller Area Networks in Autonomous Mobile Platforms. Proceedings of the 2023 26th International Symposium On Wireless Personal Multimedia Communications (WPMC).

[6] Celdrán A.H., Sánchez P.M.S., Castillo M.A., Bovet G., Pérez G.M., Stiller B. (2023). Intelligent and behavioral-based detection of malware in IoT spectrum sensors. Int. J. Inf. Secur..

[7] Rookard C., Khojandi A. Applying Deep Reinforcement Learning for Detection of Internet-of-Things Cyber Attacks. Proceedings of the 2023 IEEE 13th Annual Computing and Communication Workshop and Conference (CCWC).

[8] Zareen F., Amador M.A.F., Karam R. (2023). Malware Detection in Embedded Devices using Artificial Hardware Immunity. Res. Sq..

[9] Oladimeji D., Rasheed A., Varol C., Baza M., Alshahrani H., Baz A. (2023). CANAttack: Assessing Vulnerabilities within Controller Area Network. Sensors.

[10] Rex A., Amar R., Hacer V., Baza M., Louanne M., Mahapatra R. Harnessing IoT Technology for the Development of Wearable Contact Tracing Solutions. Proceedings of the 2021 TRON Symposium (TRONSHOW).

[11] Rasheed A., Baza M., Badr M., Alshahrani H., Choo K. (2023). Efficient Crypto Engine for Authenticated Encryption, Data Traceability, and Replay Attack Detection over CAN Bus Network. IEEE Trans. Netw. Sci. Eng..

[12] Tamil S.C., Shanmugasundaram N. Clock-Gating Techniques: An Overview. Proceedings of the 2018 Conference on Emerging Devices and Smart Systems (ICEDSS).

[13] Shila D.M., Venugopal V. Design, implementation and security analysis of hardware Trojan threats in FPGA. Proceedings of the 2014 IEEE International Conference on Communications (ICC).

[14] Subramanian K., Venkatachalam M., Saroja M. (2021). Adaptive counter clock gated S-Box transformation based AES algorithm of low power consumption and dissipation in VLSI system design. J. Phys. Conf. Ser..

[15] National Institute of Standards and Technology (2001). Advanced Encryption Standard (AES). Federal Information Processing Standards Publication 197.

[16] Mehta D., Mady A.E.D., Boubekeur M., Shila D.M. Anomaly-based intrusion detection system for embedded devices on internet. Proceedings of the Tenth International Conference on Advances in Circuits, Electronics and Micro-electronics.

[17] Hunter J., Huber B., Kandah F. Towards feasibility of Deep-Learning based Intrusion Detection System for IoT Embedded Devices. Proceedings of the 2022 IEEE 19th Annual Consumer Communications & Networking Conference (CCNC).

[18] Emnett F., Biegel M.M. (2000). Power Reduction through RTL Clock Gating.

[19] Shinde J., Salankar S.S. Clock-gating—A power optimizing technique for VLSI circuits. Proceedings of the 2011 Annual IEEE India Conference.

[20] Wu Q., Pedram M., Wu X. (2000). Clock-gating and its application to low power design of sequential circuits. IEEE Trans. Circuits Syst. I Fundam. Theory Appl..

[21] Li H., Bhunia S., Chen Y., Vijaykumar T.N., Roy K. Deterministic clock-gating for microprocessor power reduction. Proceedings of the Ninth International Symposium on High-Performance Computer Architecture, 2003. HPCA-9 2003. Proceedings.

[22] Casillo M., Coppola S., De Santo M., Pascale F., Santonicola E. Embedded intrusion detection system for detecting attacks over CAN-BUS. Proceedings of the 2019 4th International Conference on System Reliability and Safety (ICSRS).

[23] Sayadi H., Makrani H.M., Randive O., PD S.M., Rafatirad S., Homayoun H. Customized machine learning-based hardware-assisted malware detection in embedded devices. Proceedings of the 2018 17th IEEE International Conference On Trust, Security and Privacy in Computing and Communications/12th IEEE International Conference on Big Data Science and Engineering (TrustCom/BigDataSE).

[24] Rahmatian M., Kooti H., Harris I.G., Bozorgzadeh E. (2012). Hardware-assisted detection of malicious software in embedded systems. IEEE Embed. Syst. Lett..

[25] K64 Sub-Family Reference Manual. https://www.mouser.com/datasheet/2/813/K64P144M120SF5RM-1074828.pdf.

[26] arm mbed OS. https://os.mbed.com/mbed-os/.

[27] ARMmbed/mbed-os. https://github.com/ARMmbed/mbed-os.

[28] System_MK64F12.c. https://github.com/ARMmbed/mbed-os/blob/master/targets/TARGET_Freescale/TARGET_MCUXpresso_MCUS/TARGET_MCU_K64F/device/system_MK64F12.c.

[29] Taunk K., De S., Verma S., Swetapadma A. A Brief Review of Nearest Neighbor Algorithm for Learning and Classification. Proceedings of the 2019 International Conference on Intelligent Computing and Control Systems (ICCS).

[30] Jaiswal J.K., Samikannu R. Application of Random Forest Algorithm on Feature Subset Selection and Classification and Regression. Proceedings of the 2017 World Congress on Computing and Communication Technologies (WCCCT).

[31] Yang Z., Li D. Application of Logistic Regression with Filter in Data Classification. Proceedings of the 2019 Chinese Control Conference (CCC).

[32] Charbuty B., Abdulazeez A. (2021). Classification Based on Decision Tree Algorithm for Machine Learning. J. Appl. Sci. Technol. Trends.

[33] Yang F.-J. An Implementation of Naive Bayes Classifier. Proceedings of the 2018 International Conference on Computational Science and Computational Intelligence (CSCI).

[34] Xiao M., Wang H. Fast Distributed Stochastic Gradient Descent for Big Data Classification. Proceedings of the 2021 IEEE 23rd Int Conf on High Performance Computing & Communications; 7th Int Conf on Data Science & Systems; 19th Int Conf on Smart City; 7th Int Conf on Dependability in Sensor, Cloud & Big Data Systems & Application (HPCC/DSS/SmartCity/DependSys).

